# MUSTARD—a comprehensive resource of mutation-specific therapies in cancer

**DOI:** 10.1093/database/baab042

**Published:** 2021-07-26

**Authors:** Gauri Mittal, Anu R I, Aastha Vatsyayan, Kavita Pandhare, Vinod Scaria

**Affiliations:** Indraprastha Institute of Information Technology, Okhla Industrial Estate, Phase III, Delhi 110020, India; Department of Clinical Biochemistry, MVR Cancer Center and Research Institute, CP 13/516 B, C, Vellalasseri NIT(Via), Poolacode, Kozhikode 673601, India; Cancer Biology and Therapeutics: High-Impact Cancer Research Post Graduate Program, Harvard Medical School, 4 Blackfan Circle, Boston, MA 02115, USA; Department of Genome Informatics, CSIR Institute of Genomics and Integrative Biology (CSIR-IGIB), Mathura Road, Delhi 110025, India; Academy of Scientific and Innovative Research (AcSIR), Sector 19, Kamla Nehru Nagar, Ghaziabad, UP 201002, India; Department of Genome Informatics, CSIR Institute of Genomics and Integrative Biology (CSIR-IGIB), Mathura Road, Delhi 110025, India; Department of Genome Informatics, CSIR Institute of Genomics and Integrative Biology (CSIR-IGIB), Mathura Road, Delhi 110025, India; Academy of Scientific and Innovative Research (AcSIR), Sector 19, Kamla Nehru Nagar, Ghaziabad, UP 201002, India

## Abstract

The steady increase in global cancer burden has fuelled the development of several modes of treatment for the disease. In the presence of an actionable mutation, targeted therapies offer a method to selectively attack cancer cells, increasing overall efficacy and reducing harmful side effects. However, different drug molecules are in different stages of development, with new molecules obtaining approvals from regulatory agencies each year. To augment clinical impact, it is important that this information reaches clinicians, patients and researchers swiftly and in a structured, well-annotated manner. To this end, we have developed Mutation-Specific Therapies Resource and Database in Cancer (MUSTARD), a database that is designed to be a centralized resource with diverse information such as cancer subtype, associated mutations, therapy offered and its effect observed, along with links to external resources for a more comprehensive annotation. In its current version, MUSTARD comprises over 2105 unique entries, including associations between 418 unique drug therapies, 189 cancer subtypes and 167 genes curated and annotated from over 862 different publications. To the best of our knowledge, it is the only resource that offers comprehensive information on mutation-specific, gene fusions and overexpressed gene-targeted therapies for cancer.

**Database URL**: http://clingen.igib.res.in/mustard/

## Introduction

Cancer is one of the leading causes of death globally. According to the GLOBOCAN database, cancer is estimated to contribute to 18.1 million new cases and result in a total of 9.6 million deaths in 2018 alone (1). Traditional standard-of-care, namely, surgery, chemotherapy and radiation therapy target all rapidly dividing cells in the body including cells physiologically dividing at rapid rates, contributing to adverse reactions or side effects involving different organ systems like bone marrow, digestive tract, hair follicles and heart (2). Targeted therapy is a form of treatment that focuses on cells with molecular changes that are specific to a particular form of cancer. These therapies typically block the proliferation of cancer cells, induce autophagy or apoptosis or promote cell cycle inhibition and are effective in specific molecular backgrounds thereby minimizing off-target side-effects to normal tissues (3, 4). Targeted therapy is often provided in conjunction with radiation and chemotherapy.

Targeted therapy currently involves the use of small molecule inhibitors—mainly protein inhibitors, and monoclonal antibodies. Small molecule inhibitors typically target molecules important for cancer cell proliferation, angiogenesis or metastasis. An example would be imatinib mesylate (Gleevec, Novartis) that targets the Philadelphia chromosome (BCR-ABL fusion) protein in chronic myeloid leukemia (CML) patients, competitively binding to its Adenosine triphosphate (ATP) domain and disrupting the tyrosine kinase activity (2). Monoclonal antibodies can be used as therapeutic agents or for the delivery of active therapeutics, prodrug activation enzymes and chemotherapy toxins. An example would be Bevacizumab (Avastin, Genentech) that targets the glycoprotein Vascular Endothelial Growth Factor (VEGF), dysregulation of which leads to angiogenesis in metastatic breast cancer, colorectal cancer and Non-small cell lung cancer (NSCLC) (3).

Mutations play an important role in targeted therapy—they may either confer increased or decreased drug sensitivity or therapeutic resistance. The efficacy of therapy can therefore heavily depend on the tumour mutation profile (4). Thus, efforts are being made to create targeted therapies that are mutation specific. Several such drugs have been approved by the U.S. Food and Drug Administration (FDA), with many in the clinical or preclinical stage. Gefitinib is a tyrosine kinase inhibitor that has been widely prescribed for *EGFR* mutant non-small cell cancers wherein the drug inhibits phosphorylation of and enzyme activity of ATP-binding domain of the receptor via competitive inhibition (5). Recently, in January 2020, the US-FDA approved Ayvakit (avapritinib) for the treatment of unresectable or metastatic gastrointestinal stromal tumour (GIST) with a PDGFRA exon 18 mutation, including the common D842V mutation (6). A GIST harbouring this mutation usually does not respond to standard therapies (6, 7).

Given the important role cancer mutations play in shaping treatment, prognosis and overall survival, it is imperative that all available, constantly changing information be accessible in an annotated, updated format in a single compendium. Several databases have curated information about various aspects of therapy, clinical trial results, drug molecules and their target biomolecules etc. However, to the best of our knowledge, no database annotates information in a mutation-specific approach, limiting the ready use of the information by researchers and clinicians.

In this manuscript, we describe the Mutation-Specific Therapies Resource and Database in Cancer (MUSTARD) resource that provides systematic searchable access to an extensive corpus of information on mutations, gene fusions, overexpressed genes and cancer therapies targeted specifically against them, along with their intended effect and catalogued based on the evidence associated with them. To the best of our knowledge, MUSTARD is one of the most comprehensive resources systematically providing information on targeted therapies in cancer, in a clinically comprehensive approach.

## Materials and methods

### Data curation from published peer reviewed literature

An extensive literature survey using keywords combining ‘drug name’, ‘cancer-type’ and ‘genetic mutation’ from PubMed and Google Scholar was performed to retrieve an exhaustive list of publications. An exhaustive list of 2165 entries comprising 505 targeted drugs, 161 cancer subtypes and 187 genes were obtained from these publications. After filtering for duplicates and reclassifying cancer subtypes, the final dataset encompassed 2105 entries with 418 drugs, 189 cancer subtypes and 167 genes.

Additionally data were retrieved from several databases including the US-FDA, CKB (8, 9) and Clinical Interpretation of Variants in Cancer (CIViC) (10) that were used to appropriately link the data to the relevant resources as well as served as the independent evidence to support the classification. The cancer names were also normalised across the database and additionally, the evidence was classified systematically into classes based on the relevance of evidence into broad classifications as follows: Preclinical, Clinical Study, Guideline, Phase 0, Phase I, Phase II, Phase III.

### Quality control and compilation of annotations

Each of the curated data points and evidence was independently reviewed and manually annotated. The same was subject to rigorous quality check for any errors. The entire dataset was further reviewed and further cross checked. Only the consensus data were included in the final version of the compendium. Additionally, data were retrieved for each of the drug–gene/variant pair from CIViC, OncoKB (11), CKB, Precision Medicine Knowledgebase (PMKB) (12) databases as well as selected publications from PubMed database. Data retrieved included gene mutation/gene fusion, rsID, chromosome position, reference and variant allele.

**Figure 1.**
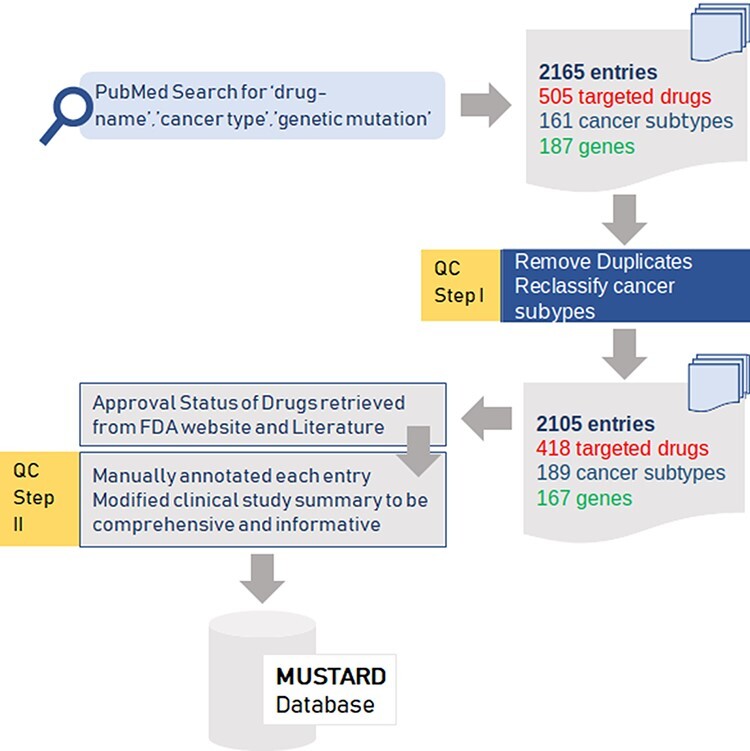
Summary of the data collection and quality control (QC) for the data for mutation-specific therapies in cancer.

The various steps in the data curation, quality control and annotation of variants are summarised as a schematic in Figure 1.

### Database and web interface design

The database search interface was designed keeping in mind the necessity to enable users to easily explore variants, common variant IDs, drugs, genes or type of evidence. The curated data were transformed into JavaScript Object Notation format and ported onto MongoDB 3.4.10, a popular open-source NoSQL database system. MongoDB v3.4.10 was used to keep track of data processing through the web interface. The data can be accessed through a web interface running on an Apache HTTP server using PHP 7.0.

The user-friendly web interface for querying the database was coded in PHP 7.0, AngularJS, HTML, Bootstrap 4 and CSS.

### Analysis of NSCLC mutations as a proof of principle application of the compendium

To validate the impact of our resource, we studied non-small cell lung cancer (NSCLC) data from The Cancer Genome Atlas (TCGA) program’s database (13) through the cBioPortal for cancer genomics resource (14). Twelve non-overlapping studies comprising 4356 samples for NSCLC (15–20), lung adenocarcinoma (20–23), (TCGA, Firehose Legacy) and lung squamous cell carcinoma (TCGA, Firehose Legacy) were obtained. The summary of datasets used is presented in Supplementary Table S1. Further, data for 34 gene variants associated with targeted therapies in NSCLC reported in our database were extracted. These genes included *ALK, KRAS, EGFR, ERBB3, ROS1, MET, BRAF, ERBB2, DDR2, KIT, TP53, STK11, NTRK1, JAK1, GNAS, PBK, RET, ABCB1, ERCC2, AURKA, RRM1, ETS2, XRCC1, FGFR1, RB1, WEE1, HAVCR2, PIK3CA, TYMS, ARAF, NRAS, PDGFRA, RASA1* and *MAPK1*. The variants reported were mapped against our dataset using bespoke scripts and proportions of patients with at least one variant associated with the clinical guidelines or FDA approved therapy in each of the dataset were compiled.

## Results and discussion

### Data compilation

The data compilation encompasses a total of 2105 entries including associations between the combinations of 418 unique target molecules, 189 cancer subtypes and 167 unique genes, and brief clinical summaries. The data were curated from a total of 862 publications and included 712 unique mutations. Of these, 1811 mutations were categorized as somatic and 132 as germline variants. Each of the entries was classified into a class based on the clinical study and type of trial as evidenced from the publications as well as database entries.

**Figure 2.**
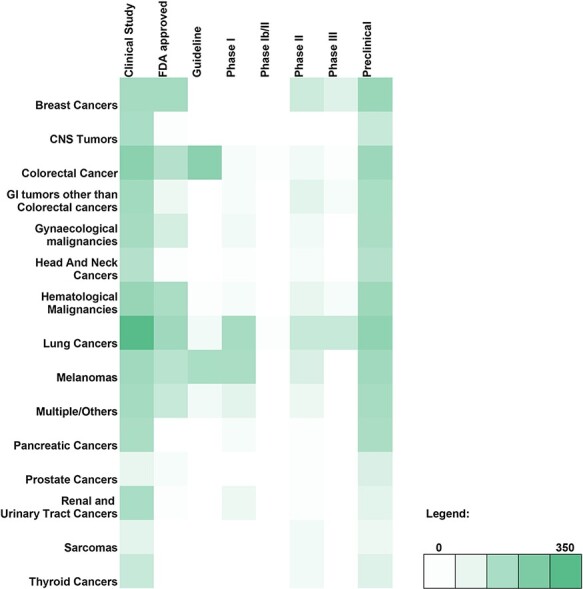
Heatmap depicting the genomic variant and therapy combinations studied for different types of cancer.

A breakdown of the number of genetic variant-therapy combinations classified by broad cancer type and evidence class is shown in the form of a heatmap (Figure 2). Of the 2100 unique entries, 2062 were broadly categorised on the basis of the type of cancer studied. The remaining 38 included studies involving only cell lines and were therefore excluded. The largest number of variants observed was for lung cancer, which encompassed 29.43% of all variant-therapy combinations. Treatment guidelines for actionable targets in thyroid cancers (1.04%) and sarcomas (0.67%) are close to none, pointing to an unmet need in targeted therapy for these cancers.

The variants were also analysed with respect to therapies. At least one mutation-specific therapy was available for 712 unique variants, of which BRAF-V600E had the maximum number of evidence annotations, amounting to a total of 64 annotations for 28 unique therapies or combinations thereof. Ten of these are catalogued FDA-approved and seven guidelines, while 47 are in different stages of study. These form 3% of all annotations, suggesting the widespread application of the therapy in multiple cancers including colorectal, melanoma, ovarian and NSCLC and also rightly suggested in clinical guidelines. The database includes 77 entries for unique gene fusions and their respective targeted therapies including 52 drugs and 26 entries for overexpressed proteins against 28 matched targeted molecular therapies.

**Figure 3.**
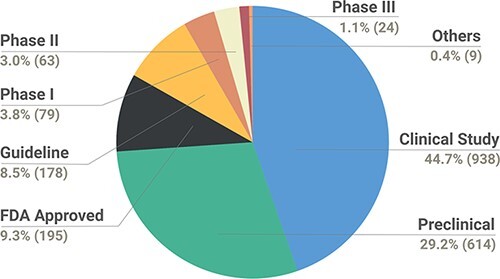
Pie chart depicting the breakdown of types of studies reported in the database.

The data were also catalogued by the relevance of the study as a preclinical/clinical observation to aid the interpretation of evidence. In total, the data collection encompasses 938 variants from clinical study, 614 from preclinical, 195 FDA approved, 178 guidelines, and 79, 63 and 24 Phase I, II and III studies. In addition, the data collection encompass nine variants collectively termed ‘Others’, which are a part of preclinical (cell line xenograft, cell culture and patient cell culture), Phase 0 or Phase Ib/II studies. The types of studies reported in the database are depicted in Figure 3.

The database also indexes 174 annotations from clinical guidelines, including 14 derived from National Comprehensive Cancer Network, 145 from PMKB and 15 from the CIViC database.

### Database and web interface features

The database is designed to have a user-friendly interface to enable easy access to clinicians and researchers. The interface allows the user to query the database or browse the database based on the annotations including clinical evidence types. The database is accessible at http://clingen.igib.res.in/mustard/

**Figure 4.**
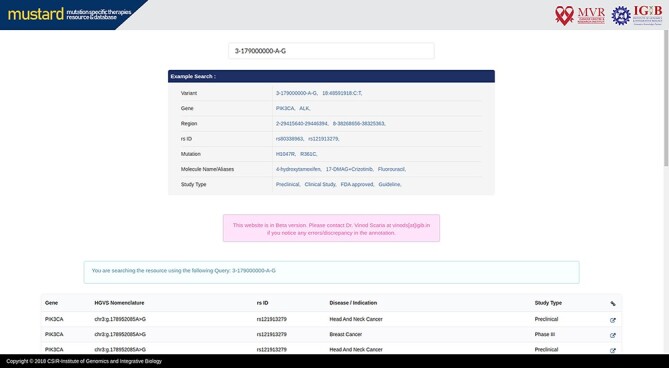
MUSTARD homepage showcasing the different search query formats that can be used.

**Figure 5.**
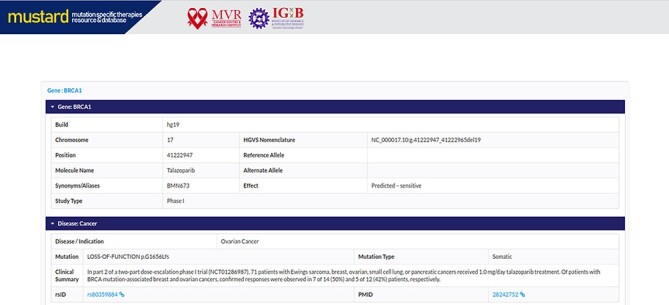
Screenshot of the MUSTARD database showing the expanded search results.

Users can search the resource in multiple query formats, including variant name, molecular designation of the drug, study type, genomic location, rsID or gene name. The result page will link further information to the variant selected, including a complete genomic description of the mutation, therapies and their effects studied. A complete list of example formats is available on the homepage (Figure 4). The comprehensive expanded result page is captured in Figure 5.

The resource also enables users to browse the database based on the clinical evidence available for mutation-specific therapies. Presently, the evidence types that could be browsed are categorised as preclinical, clinical study and guideline. In addition, users can also browse evidence for mutation-specific therapies that are US-FDA approved.

### Comparison with other resources

Given the importance of targeted therapies in cancer, it is essential to have easy access to systematically curated and annotated published therapy data. However, to the best of our knowledge, no other resource addresses this concern adequately. For example, ClinVar (24), which offers extensive information about variants, now offers external link-outs to The Drug Gene Interaction Database (DGIdb) (25). However, the DGIdb does not provide any information regarding the druggability of specific mutations (25, 26). Similarly, PharmGKB (27) focuses on potentially clinically actionable gene-drug associations and genotype-phenotype relationships but not mutation-specific therapies. The Cancer Molecular-Targeted Therapy database (CMTTdb) (28) offers information regarding anticancer therapy agents and their molecular targets (e.g. receptors) but does not discuss the role mutations play. The Therapeutic Target Database (TTD) (29) and Drugbank (30) offer similar information along the same lines for a wider variety of disorders, while GtoPdb (IUPHAR/BPS Guide to PHARMACOLOGY) (31) offers information about interactions between drugs and their targets in the human genome; none of these resources offer information regarding mutation-specific therapies in cancer. This information has been summarized in Supplementary Table S2. A Venn diagram representing the overlap between the variants in the different databases is shown in Supplementary Figure S1.

### Utility of data collection in the understanding prevalence of genomic variants associated with mutation-specific therapies

To explore whether the compendium of variants annotated could be used to understand the genetic epidemiology of targeted therapies from genomic datasets, we used a publicly available dataset of genomic sequences generated and available in public domain for NSCLC. The choice of the cancer was prompted by the fact that a number of publications from across the world reported variants and targeted therapies for NSCLC.

We queried data from 4356 samples of 3952 patients encompassing 12 non-overlapping studies summarised in Supplementary Table S1. In addition to the studies mentioned in the table, TCGA Firehose Legacy Lung Squamous Cell Carcinoma and Adenocarcinoma raw data at the NCI were also included in the analysis. Our analysis of genomic variants in 34 genes (*ALK, KRAS, EGFR, ERBB3, ROS1, MET, BRAF, ERBB2, DDR2, KIT, TP53, STK11, NTRK1, JAK1, GNAS, PBK, RET, ABCB1, ERCC2, AURKA, RRM1, ETS2, XRCC1, FGFR1, RB1, WEE1, HAVCR2, PIK3CA, TYMS, ARAF, NRAS, PDGFRA, RASA1, MAPK1*) reported in our database for NSCLC was performed as detailed in the Materials and Methods section. We obtained a total of 6255 unique samples from 11 of the 12 studies. These comprised of 2597 unique variants from 2845 unique sample IDs.

**Figure 6.**
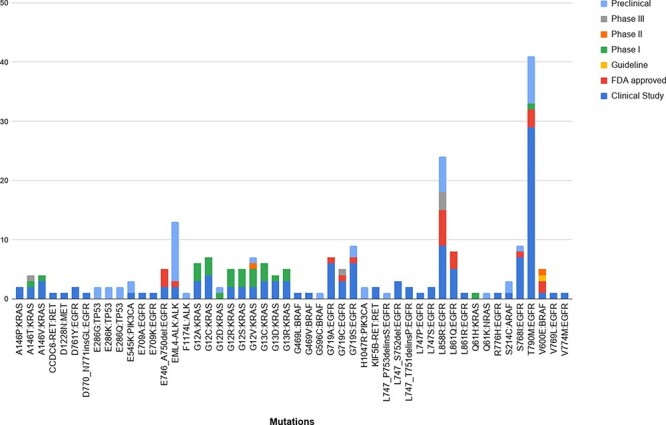
The breakdown of the 50 unique variants overlapping between the patient dataset and MUSTARD, according to their stage of study as per the database annotations.

Upon mapping the variants obtained from the studies against the ones reported in our database, we obtained 96 unique variants that were common to both. Of these, 50 unique variants were linked to NSCLC. Further analysis of these variants revealed that 124 of the common therapies were in the clinical study stage, 45 were preclinical, 22 were FDA approved, 1 was a guideline, and 26, 2 and 5 belonged to Phase I, II and II, respectively. Figure 6 summarizes the breakdown between the variants and their stage of study.

**Figure 7.**
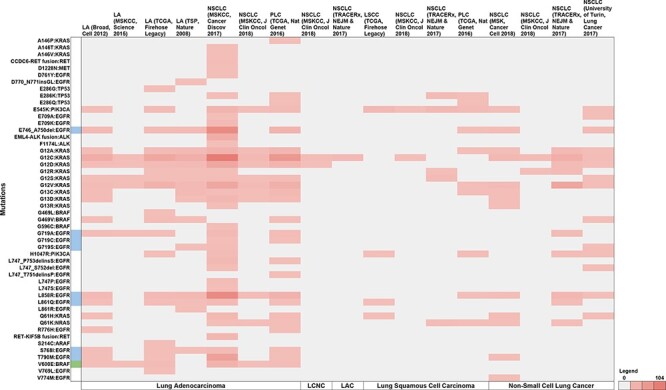
Summary of the breakdown of each of the 50 unique variants across the 11 studies. The variants highlighted in blue represent molecules that have been FDA approved, while green represents guidelines as well as FDA approved molecules. Note: LA—Lung adenocarcinoma, NSCLC—non-small cell lung cancer, PLC—pan-lung cancer, LSCC—lung squamous cell carcinoma, LCNC—large cell neuroendocrine carcinoma, LAC—lung adenosquamous carcinoma.

Of the 12 TCGA studies, non-small cell cancer (MSKCC, Cancer Discov 2017) reported the highest proportion of patients with NSCLC variants (43.9%), followed by pan-lung cancer (TCGA, Nat Genet 2016) at 16.2%, NSCLC (TRACERx, NEJM & Nature 2017) at 10.5%, lung adenocarcinoma (TCGA, Firehose Legacy) at 8.21% and lung adenocarcinoma (TSP, Nature 2008) at 6%, respectively. The lowest proportion of patients were reported by lung adenocarcinoma (MSKCC, Science 2015) at 0.76%. Further, no non-overlapping data were found in the NSCLC (MSK, Science 2015) study, which was therefore excluded from representation. Figure 7 summarises the study-wise breakdown of each of the 50 unique variants.

Across the 11 studies, 1221 unique patient IDs reported 50 unique mutations in 10 genes (*EGFR, KRAS, BRAF, TP53, ALK, PIK3CA, RET, ARAF, MET*, and *NRAS*), and were linked with five NSCLC types (lung adenocarcinoma, lung squamous cell carcinoma, NSCLC, lung adenosquamous carcinoma and large cell neuroendocrine carcinoma). We thus show that such a compilation could effectively estimate the prevalence of variants for which targeted therapies are available from public domain datasets.

## Conclusion

In summary, MUSTARD is a powerful resource that can help bring together published information to clinicians and researchers, in a curated and well-annotated form. The database is unique as it compiles actionable mutations, gene fusions and overexpressed proteins with available targeted therapies, clinical trial links, study summary and predicted effect of the drug under a single umbrella that would enable translating the information in a clinic/out-patient department setting while deciding treatment for a patient. With this resource, we hope to bridge the gap between the literature and real-world application of the published information, hopefully driving clinical implications in a positive direction.

## Supplementary Material

baab042_Supp

## Data Availability

Data sharing is not applicable to this article as no new data were created or analysed in this study.
