# Supplementary figures and images for: MUSTARD—a comprehensive resource of mutation-specific therapies in cancer
Source: Database (Oxford). 2021 Jul 26;2021:baab042. doi: 10.1093/database/baab042 (PMC8312254; doi:10.1093/database/baab042)

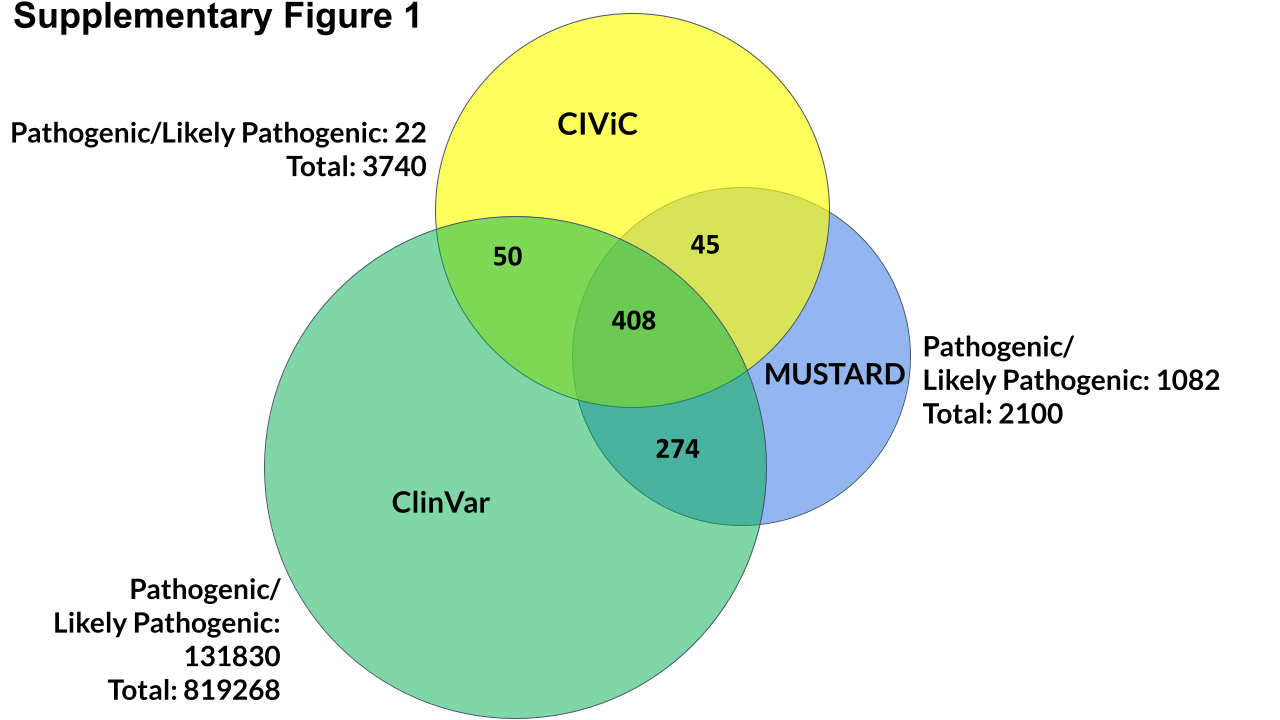

Supplement: baab042_Supp [file baab042_supp.zip › supplementary_figure1.png]
